# Key factors for national spread and scale-up of an eConsult innovation

**DOI:** 10.1186/s12961-020-00574-0

**Published:** 2020-06-03

**Authors:** Isabella Moroz, Douglas Archibald, Mylaine Breton, Elizabeth Cote-Boileau, Lois Crowe, Tanya Horsley, Lirjie Hyseni, Gina Johar, Erin Keely, Katharina Kovacs Burns, Craig Kuziemsky, Jim Laplante, Ariana Mihan, Luis Oppenheimer, Don Sturge, Delphine S. Tuot, Clare Liddy

**Affiliations:** 1 C.T. Lamont Primary Health Care Research Centre, Department of Family Medicine, University of Ottawa, Ottawa, Canada; 2 Bruyère Research Institute, Ottawa, Canada; 3 Faculty of Medicine and Health Sciences Research, University of Sherbrooke, Longueuil, Canada; 4 Royal College of Physicians and Surgeons of Canada, Ottawa, Canada; 5 eHealth Centre of Excellence, Waterloo, Canada; 6 South East Local Health Integration Network, Belleville, Canada; 7 Department of Medicine, University of Ottawa, Ottawa, Canada; 8 Division of Endocrinology/Metabolism, The Ottawa Hospital, Ottawa, Canada; 9 Ontario eConsult Centre of Excellence, The Ottawa Hospital, Ottawa, Canada; 10 Alberta Health Services, Edmonton, Canada; 11 MacEwan University, Edmonton, Canada; 12 Care Partner, Ottawa, Canada; 13 Department of Surgery, University of Manitoba, Winnipeg, Canada; 14 Central Health, Gander, Canada; 15 Division of Nephrology, University of California San Francisco, San Francisco, United States of America; 16 Center for Innovation in Access and Quality, University of California San Francisco, San Francisco, United States of America

**Keywords:** Innovation, spread and scale-up, electronic consultation, eConsult, policy

## Abstract

**Background:**

Expanding healthcare innovations from the local to national level is a complex pursuit requiring careful assessment of all relevant factors. In this study (a component of a larger eConsult programme of research), we aimed to identify the key factors involved in the spread and scale-up of a successful regional eConsult model across Canada.

**Methods:**

We conducted a constant comparative thematic analysis of stakeholder discussions captured during a full-day National eConsult Forum meeting held in Ottawa, Canada, on 11 December 2017. Sixty-four participants attended, representing provincial and territorial governments, national organisations, healthcare providers, researchers and patients. Proceedings were recorded, transcribed and underwent qualitative analysis using the Framework for Applied Policy Research.

**Results:**

This study identified four main themes that were critical to support the intentional efforts to spread and scale-up eConsult across Canada, namely (1) identifying population care needs and access problems, (2) engaging stakeholders who were willing to roll up their sleeves and take action, (3) building on current strategies and policies, and (4) measuring and communicating outcomes.

**Conclusions:**

Efforts to promote innovation in healthcare are more likely to succeed if they are based on an understanding of the forces that drive the spread and scale-up of innovation. Further research is needed to develop and strengthen the conceptual and applied foundations of the spread and scale-up of healthcare innovations, especially in the context of emergent learning health systems across Canada and beyond.

## Introduction

Expanding healthcare innovations from the local to national level is a complex pursuit, particularly for projects pertaining to eHealth services. Although research in the area of spread and scale-up of healthcare innovations has been quite limited, a seminal body of work on diffusion of innovation by Rogers [1] identified the primary determinants of success in the spread of new ideas, namely innovation attributes (relative advantage, compatibility, complexity, trialability and observability), communication channels, characteristics of adopters (innovators, early adopters, early majority, late majority and laggers), and social system within which the diffusion of innovations takes place.

Despite the widespread acceptance of this theory, accumulating evidence shows that “*diffusion is an atypical outcome, since the vast majority of innovations fail to diffuse*” [2]. Analyses of unsuccessful efforts to scale-up have identified common challenges, which include underestimating the resources required for scale-up, failure to understand the importance of politics and policy in successful scale-up, not considering the conditions needed for scale-up early in the process of innovation development, and an overemphasis on either the vertical (institutionalisation through policy or legal action) or horizontal (replication or expansion) spread of innovations as opposed to considering both [3]. A study of non-adoption and abandonment of innovations found that limited success in attempts to scale-up, spread or sustain the programme was explained, in many instances, by the tendency to assume that the issues to be addressed were simple or uncomplicated (hence knowable, predictable and controllable) rather than complex (i.e. inherently unknowable, unpredictable, dynamic and emergent) [4]. The authors of this study emphasised that the realities of dealing with the multiple complexities of healthcare require a contextualised and adaptive approach that can evolve with a rapidly shifting policy context or continued evolution of the technology.

Such complexities are especially evident in the Canadian context. In addition to its enormous size and geographically dispersed population, Canada presents challenges to eHealth innovators due to its fragmented healthcare system, which consists of 13 unique provincial and territorial jurisdictions [5]. Healthcare budgets are legislated at the provincial level and nearly every component of the healthcare system is provincially controlled, including regulation, payment, human resources, programming and IT infrastructure. Communication between provinces is limited and there is little to no capacity for sharing or distribution of innovations. These challenges have caused Canada to gain a reputation as a “*land of perpetual pilot projects*”, where promising solutions fail to gain traction beyond the regional level [6].

Despite these challenges, a few services have managed to avoid being relegated to the ‘land of perpetual pilot projects’ and demonstrated a capacity for expansion to new jurisdictions within and between provinces. One such example is the Champlain BASE™ (Building Access to Specialists through eConsultation) eConsult service, a secure online platform that allows primary care providers (PCP) and specialists to communicate regarding a patient’s care. PCPs log into the service using any device with a web browser, enter a clinical question regarding their patient and submit it to a chosen specialty group. The case is assigned to a specialist from the chosen group based on availability, who responds within 1 week with advice on the patient’s care, a recommendation for referral or a request for further information. Specialists respond to cases in a median of 0.9 days and two-thirds of patients receive care without needing a face-to-face specialist visit [7]. First launched in 2009, this innovative service has grown from a small proof-of-concept to a fully implemented regional service, with a solid body of evidence supporting its ability to improve access to specialist advice, reduce wait times, lower costs, and deliver high levels of patient and provider satisfaction [8–10].

Building on eConsult’s regional success, our team sought to expand the eConsult service through both spread (i.e. adoption or replication in other regions of other Canadian provinces) and scale-up (i.e. expansion from the regional to provincial level in Ontario). As part of this process, we held a national Policy Forum, to which we invited a diverse group of stakeholders from across Canada. Our intent was to (1) explore the existing research evidence and knowledge gaps related to eConsult’s spread and scale-up in Canada, (2) identify the best ways to adopt patient-centred approaches throughout the spread and scale-up process, and (3) influence eConsult development strategies.

In this paper, we aim to identify the key factors emerging from the Policy Forum that support eConsult’s spread and scale-up across Canada.

## Methods

### Design

We conducted a qualitative content analysis to identify key factors associated with eConsult’s potential spread and scale-up. While these two terms are often used interchangeably, spread and scale-up refer to different processes [11]. Spread refers to the organic procession of an innovation’s diffusion to new jurisdictions [12], while scale-up involves the deliberate adoption of an existing service at a higher policy level (e.g. from regional to provincial) to reach more patients and foster policy development [13]. A common analogy is that spread consists of horizontal expansion across different jurisdictions, while scale-up refers to vertical expansion across different levels of government. In eConsult’s case, spread involves the service’s replication by innovators in other provinces or territories, while scale-up involves its adoption in Ontario at the provincial level.

### Setting

Our team held a 1-day national eConsult Policy Forum in Ottawa, Canada, on 11 December 2017. The meeting served as a follow-up to an initial policy-focused meeting — the National eConsult Policy Think Tank — that took place in Ottawa on 5 December 2016. The Think Tank yielded specific, actionable recommendations regarding provincial and national policy related to eConsult, particularly in the areas of interjurisdictional licensing, payment, privacy, quality assurance and regulation [14].

The Policy Forum started with brief presentations by eConsult researchers and regional implementation partners, who reported on progress made over the past year in eConsult implementation on provincial and national levels (see Additional file 1 for a meeting agenda), followed by a panel discussion by patients reflecting on their experiences with eConsult. Next, participants attended small group sessions facilitated by multiple eConsult leaders, which focused on issues relevant to national expansion. The afternoon portion consisted of two sets of small group sessions — (1) ‘tabletop sessions’, which focused on the experiences, knowledge and tools necessary to spread and scale eConsult, and (2) ‘hot topics – policy briefs’, during which participants debated the key policy issues summarised in the five policy briefs arising from the 2016 Think Tank [14, 15]. The meeting concluded with a panel of eConsult national leaders’ reflections on the day.

### Participants

We emailed meeting invitations to 103 individuals from key stakeholder groups, including representatives from provincial and territorial governments, national and provincial organisations, healthcare providers, researchers, and patients, who had expressed an interest in eConsult’s spread and scale-up. The Ottawa Hospital — a regional partner of Champlain BASE^TM^ eConsult service — agreed to co-host the meeting.

### Data collection

All plenary and small working group sessions, which ranged from 30 to 60 minutes in duration, were audio-recorded, yielding a total of 19 recordings. Recordings were transcribed and imported into NVivo 11 software to enable qualitative content analysis.

### Data analysis

The Framework for Applied Policy Research was used to guide the content analysis [16]. It consists of five steps: familiarisation, identification of a thematic framework, indexing, charting, and mapping and interpretation.

#### Step 1: Familiarisation

One of the reviewers (AM) read a selection of the available transcripts, consisting of the patient panel reflections and report-back forms from three main sessions. These transcripts were chosen as they represented summaries of all topics discussed at the meeting. AM made notes on the key ideas and recurrent themes that emerged from these transcripts (e.g. patient engagement, partnerships and governance).

#### Step 2: Identification of a thematic framework

We considered several frameworks relating to the spread and scale-up of health interventions that could be applied to our data [13, 17–20]. Ultimately, we selected a thematic framework created by Charif et al. [17] since it was specific to primary care interventions and identified the following four key components of scaling up evidence-based practices in primary care: human resources (e.g. policy-makers/managers, providers, community healthcare workers), healthcare infrastructure, policy/regulation, and financing. These dimensions were highly aligned with the initial themes identified in the familiarisation step and relevant to our research question. We expanded the framework dimensions to include ‘patients’ based on our previous work, where active participation of patient partners transitioned the discussions and policy recommendations for the national spread and scale-up of eConsult from provider-centred to patient-centred thinking [14]. The initial codebook was established by applying the themes that emerged in the familiarisation phase to the Charif et al. [17] framework.

#### Step 3: Indexing

Indexing involved identifying portions or sections of the transcribed data that corresponded to particular themes. Given the structure of the Forum, not all of the recorded sessions provided novel information related to spread and scale-up. For instance, the Forum began with presentations from different stakeholders discussing the service’s projects in their region, which served to provide context to attendees but involved no discussion of policy. Eight transcripts were excluded from the analysis for this reason, resulting in 11 transcripts included in the final sample. To ensure rigor and reduce the risk of selection bias, all excluded transcripts were thoroughly read by one reviewer (AM) to look for outliers and any disconfirming evidence. A complete list of transcripts is available in Additional file 2.

Two reviewers (AM and IM) independently coded three transcripts using the initial codebook, meeting regularly to discuss any discrepancies and revise the codebook accordingly. This process was repeated until seven transcripts had been coded, at which point AM and IM submitted the codebook to a national working group consisting of 14 self-selected Forum attendees (CL, CK, DA, DT, DS, EC, EK, GJ, JL, KKB, LC, LH, LO, MB, TH). Working group participants came from diverse disciplinary backgrounds, including public policy research, business, medical doctors, representatives of regulatory colleges and patient partners. Using a participatory approach, engagement of Forum participants in the coding of the data helped to verify interpretation and increase validity of the data analysis.

The working group members met via teleconference to discuss the approach to the analysis and review the initial findings. After the meeting, the working group members provided additional feedback and comments. Core members of the working group (CL, EK, AM, IM) held an additional meeting to reflect on the revised codebook in more depth. Upon careful consideration, it was determined that the original framework by Charif et al. [17] needed further adaptation following the addition of ‘patients’ to more appropriately align with the themes emerging from the data (e.g. financing was deleted as it was not coming out as a theme from the data). The codebook was revised to better reflect the adaptation and the key themes and sent to the working group members for review. There were no objections to the revisions.

#### Step 4: Charting

AM sent a revised codebook to members of the working group for in-depth review. Each member was assigned two themes to review and discuss with the group at the next meeting. Members of the working group highlighted a few minor areas for adjustment, identified key quotations and confirmed that data saturation was likely reached.

#### Step 5: Mapping and interpretation

AM and IM revised the codebook based on comments from the working group and circulated it for review at a final meeting held on 21 August 2018, during which the consensus on the final codebook was obtained.

## Results

A total of 64 of 103 invited participants (62% of those invited) attended the meeting. Among attendees, 22% self-identified primarily as administrators, 16% as general public, including patient partners, 36% as healthcare professionals and physicians (including fellows), 14% as non-healthcare (e.g. government officials), 7% as researchers (including PhD students), and 5% as sponsor (partner organisation) representatives.

Four main themes emerged from our analysis, namely (1) population care needs and access, (2) stakeholders and their roles, (3) building on current strategies and policies, and (4) measuring and communicating outcomes. A summary of the key points for each theme is available in Table 1.

**Table 1:** Key points associated with the four themes emerging from the analysis of the Policy Forum

Theme	Key Points
Identifying population care needs and access problems	• Keep needs of the target population central to any efforts to spread and scale-up eConsult • Ensure service does not overlook patients with complex circumstances (e.g. rural areas, indigenous communities) who often face inequitable access to care
Engaging stakeholders	• Collaborate with stakeholders from a variety of disciplines and organisations • Establish clear governance and accountability • Partner with national and provincial organisations (e.g. professional colleges)
Building on current strategies and policies	• Align with existing government priorities (e.g. Health Links, opioid strategy) • Adapt current clinical and operational models • Partner with existing telemedicine organisations • Address key considerations of privacy, licensing and regulatory differences between jurisdictions • Develop strategies for quality assurance, improving technology, and adopting clinician and practice workflows
Measuring and communicating outcomes	• Develop an evaluation system that includes measures relevant to physicians, administrators and patients • Emphasise benefits of eConsult beyond improving access or reducing wait times (e.g. educational value) • Include eConsult in medical curriculum as a standard of practice

### Identifying population care needs and access problems

Participants discussed the importance of keeping the needs of the target population central to any efforts to spread and scale-up eConsult. Specifically, participants described excessive wait times as a significant barrier to accessing care, which eConsult had the potential to address:

“*I didn’t realise how bad wait times are in Canada. These are atrocious* […] *I’ve been fortunate to visit other countries and they just cannot believe the wait times in this country* […] *We’re doing other things now. It’s still a problem, and this project, fortunately, is still here and is addressing an issue that remains towards improving access for Canadians*.”

Participants identified the particular challenges of providing specialty care access to populations with complex circumstances and discussed the importance of reaching these populations with eConsult. The term ‘complex circumstances’ was used to denote not just patients with complex clinical needs, but also those who face social, economic and geographical challenges that can further impose specific barriers to accessing care:

“*There were lots of different special populations that were brought up that we need to ensure that we do enable eConsult for. So just some examples, indigenous groups, pediatric, mental health, LGBT community, rehab, folks in long-term care with a focus on dementia, frailty and of course chronic pain sufferers would also benefit from eConsult*.”

Many participants noted that patients with complex circumstances often face inequity in access and described eConsult’s ability to address this inequity as one of its chief advantages. Participants suggested emphasising the notion of eConsult “*as a right*” within its messaging:

“*The problem is that there’s no equity. So people living in rural areas or with certain types of conditions may not be able to get to the services. And therefore may not be able to benefit from the kinds of services that are available.* […] *So we have to do something to make sure that that equity is available and present*”.

Targeting rural areas was seen as particularly important. In more remote regions of the Canadian north, eConsult was seen as a necessity:

“*So eConsult to us in Nunavut is more than just nice-to-have or how convenient for us. It’s actually a necessity.* […] *we’re an extremely large jurisdiction. We cross three time zones, so we’re 25 communities across three time zones.* […] *our referral patterns run north–south, so we send patients in the eastern region to Iqaluit or Ottawa. And then our central region we go to Winnipeg, and then in our western region, we go to Yellowknife* […] *and then off to Edmonton from there. So thinking about how you implement eConsult in a jurisdiction like that is incredibly overwhelming at times*.”

Participants also recognised eConsult’s ability to provide support for PCPs caring for patients with complex circumstances and to enable interdisciplinary disease management.

### Engaging stakeholders who were willing to roll up their sleeves and take action

When discussing strategies for eConsult’s spread and scale-up, participants emphasised the importance of collaborating with stakeholders from a variety of disciplines and organisations. As one participant noted: “*This is a journey you cannot do alone. You need the collaboration of the experts in the field. You need the collaboration of the people that have the outreach. You need the local partnerships*”. Examples of stakeholders included various national and provincial organisations, patients and change champions. The term ‘change champions’ was never explicitly defined but, in the context of the meeting discussions, it was used to denote individuals who were willing to actively promote eConsult within their organisations and wielded influence across the socio-political environment outside their organisations. Change champions could include clinicians, administrators, patients and members of any other stakeholder group. In terms of patients, participants emphasised the role of engaging patients as partners in the design and promotion of eConsult:

“*You want to make sure that you engage your providers but also your patients. There was definitely feedback suggesting that patient partners are not necessarily used as well as they can be to help drive home the need for an eConsult service. So using patients and providers to help sell your message is a great and key strategy*.”

An important component of collaborating with stakeholders was establishing clear governance in order to decide what team members or groups would be responsible for making different decisions and oversee strategic and operational components of the service. As one participant stated: “*How are you going to create a structure to help you operationalise the day-to-day function? And then a strategic governance layer. How does this fit in with other strategic priorities relative to that patient population as well as digital health?*”

Governance also pertained to accountability, which affected such areas as privacy, licensing and education:

“*So, for example, from an accountability standpoint, how do you describe who the authorities are? How do you legally bind those authorities so that they are able to support the privacy and security standards within your programme? What’s the training and education requirement like?*”

Participants identified a number of national organisations with the jurisdiction and resources to oversee accountability in these issues, including the Canadian Medical Protection Association, the Royal College of Physicians and Surgeons of Canada, and the College of Family Physicians of Canada. Additionally, participants suggested that the provinces’ professional colleges could oversee licensing issues, as they already perform similar duties.

### Building on current strategies and policies

Participants emphasised the importance of building upon existing eHealth strategies to enable the spread and scale-up of eConsult. In particular, participants suggested aligning with existing government priorities (e.g. Health Links, opioid strategy) as effective strategies to introduce new healthcare delivery programmes such as eConsult:

“*Thinking about this scale and spread nationally, I think it’s important to remember to not reinvent the wheel. There’s multiple environmental scans out there from probably privacy regulation and even looking historically back on different platforms that previously were implemented within the past 5 years. And looking at the barriers and facilitators from kind of those implementations and learning from those within the implementation of the eConsult*.”

Other suggestions included adapting current clinical and operational models to support eConsult and partnering with existing telemedicine organisations. Similar suggestions were raised regarding regulation, in that existing regulatory and best practices, including templates for licensing in other jurisdictions, could be applied to eConsult.

Participants also discussed a number of known policy-level and system-level enablers to build on for a broad scale eConsult implementation. Policy-level enablers included addressing the key considerations of privacy, licensing and regulatory differences between jurisdictions. For instance, one participant suggested developing “*a single virtual license* [for eConsult] *that would cover the whole country and could be adopted or owned inside one of the larger provinces*” noting that such a body “*would be a lot more efficient way of negotiating* […] *and finalising the terms*”. System-level enablers included developing strategies for quality assurance, improving technology, and adopting clinician and practice workflows. Participants stressed the importance of making the service simple and reliable in order to encourage PCP and specialists to use it. Other effective enablers included taking a multidisciplinary approach, including a research and education focus, and capitalising on physician leadership, patient engagement, and connecting people and organisations.

### Measuring and communicating outcomes

Participants identified the ability to evaluate eConsult’s impact as another key factor in its spread and scale-up. Evaluation provides vital feedback to the individuals implementing the service and lets them clearly identify “*how we can do a better job of monitoring and evaluating what’s working, what’s not working*”. As such, participants noted that a system of evaluation should be an integral part of the service and include measures that are relevant to physicians, administrators and patients.

An example of evaluation strategies emerged during the regional updates portion of the Forum, where representatives from different regions took the opportunity to articulate the service’s impact using key measures of success (e.g. eConsult completed, providers enrolled, specialty groups available) and used these items to tell the story of the service’s adoption and offer lessons learned from the experience:

“*We started small* […] *with a handful of family physicians and a handful of specialists.* […] *to date we have 27 participating specialties and a number of subspecialties as well. We’ve got just over 160 primary care providers on board. And we’ve generated-- last night it was 799 eConsults.* […] *Which is phenomenal. We are so pleased with our growth and success*.”

With regards to communicating outcomes, participants emphasised the importance of discussing its benefits beyond improving access or reducing wait times. For instance, using eConsult for strengthening education and communication among healthcare providers was viewed as an important learning opportunity, especially for chronic pain care and geriatric care:

“*We know that our healthcare providers are not getting appropriate education in student curricula and also in continuing education. So I envision that eConsult could be incredible when it comes to getting a bunch of pain specialists together to provide education.* […] *they will learn pain management from specialists, but also they will be able to get advice on a case-by-case basis that is so helpful for them. They feel less isolated, and they can help their patient*.”

Communicating the benefits and impacts of eConsult with healthcare providers was also raised as an important point: “*I think education is a huge component of eConsult. None of us like change. It’s difficult and I think that educating GPs for all the benefits and what this can mean to them and their practice is really crucial*”. Final reflection highlighted the importance of including eConsult in medical curriculum as a standard of practice:

“*And finally, one of the key things also that came out was a really strong theme again about education. And we talked about the need to create the curriculum.* […] *So really starting to think about getting into the medical schools, but also having it as a standard – an education standard, which would then lead into a standard of practice*.”

## Discussion

This study identified four main themes that were critical to support the intentional efforts to spread and scale-up eConsult across Canada, namely (1) identifying population care needs and access problems; (2) engaging stakeholders who were willing to roll up their sleeves and take action; (3) building on current strategies and policies; and (4) measuring and communicating outcomes. We recommend that other healthcare innovators base their spread and scale efforts on consideration of these factors as outlined in the checklist in Table 2.

**Table 2:** Four-point checklist for driving the spread and scale of healthcare innovations

Factor	Questions
Population needs	□ Have you clearly identified the problem based on population needs? □ Does your solution address the problem?
Stakeholder engagement	□ Are your stakeholders engaged and in a position to take action? □ Have you included patient partners?
Existing policies and strategies	□ Have you considered the existing strategies and policies to build on?
Measurement and communication	□ Have you embedded measurement and continuous quality improvement into the process?

In the theme ‘population care needs and access’, participants reinforced the importance of ensuring that the proposed solution (i.e. eConsult) matches the problems affecting the target community. In our study, eConsult was seen as a necessity rather than a mere convenience for patients, as it is recognised for its ability to enable equitable access to care across populations and regions. Specifically, participants emphasised eConsult’s ability to enhance timely access to quality care for the disadvantaged patients with complex clinical needs and those facing complex social, economic and geographical conditions. A growing body of international evidence supports this notion. In the Los Angeles County Department of Health Services, a rapid adoption of an eConsult system over a 4-year period dramatically improved access to specialist advice for a large disadvantaged population with historically poor access to specialty care [21]. Similarly, a store-and-forward telemedicine tool created by Médecins Sans Frontières has been shown to improve the primary-specialty care interface in low-resource settings, allowing field doctors to obtain an expert opinion within a few hours, wherever they are located in the world [22]. In our own Canadian setting, disparity of access to care and inappropriately long wait times are the most concerning issues that threaten patient safety [23–26]. Thus, as regions seek to spread eConsult, a suggested first step is to identify local needs through wait time studies and engagement of the patient and provider community to identify priority areas specific to that region. Simply implementing a ‘cookie-cutter’ approach that worked in another region is unlikely to yield the same results without this step.

In the ‘stakeholder engagement’ theme, participants highlighted the importance of identifying and engaging individuals who can influence change, with specific emphasis on patient partners and those within organisations that will end up playing a significant role in the service’s governance, development and implementation. The notion of change champions came up repeatedly in the discussions. The concept of change champions is not new; they have been typically described as important for moving new innovations through the phases of initiation, development and implementation [27], thus playing a key role in the successful scale-up of health service innovations [28]. A change champion can be a dedicated person or an organisation with the motivation and means to encourage, guide and support widespread adoption. In our experience, having engaged patient partners on our team likely served as the catalyst for building collective capacity and motivating other stakeholders to take action. In our earlier work, we found that active participation among our patient partners helped shift our focus from provider-centred to patient-centred thinking around relevant policy issues [15]. However, this finding raises the question of what is the role of change champions in sustaining eConsult, in addition to its spread and scale. This finding explicitly demonstrates the importance of involving patients and caregivers in healthcare policy decision-making to support the scale and spread of eConsult and similar technologies.

The theme of ‘building on current strategies and policies’ encompassed suggestions to harness policy, system and implementation enablers by applying existing regulatory and best-practice guidelines to eConsult and aligning them with existing government and population health priorities. Participants emphasised the importance of understanding the current context in which the service is being implemented, as in many cases there are already policies or programmes in place that could be adopted by or adapted to eConsult, allowing innovators to avoid ‘reinventing the wheel’. This advice aligns with recommendations from the Advisory Panel on Healthcare Innovations, which noted that positive changes in Canadian healthcare systems could be accelerated by mechanisms that challenge our propensity to reinvent the healthcare wheel city by city and region by region [29]. The evidence shows that, while the current institutionalisation of models and scopes of practice has shielded the system from radical reform, there have been incremental changes across micro, meso and macro levels to address specific populations with higher needs or access issues. Examples include the development of new roles, such as patient navigators and pharmacy technicians, and the expansion of scopes of practice for professions such as nurse practitioners and pharmacists. Innovative solutions must often work around structural barriers unless integrated into the health system transformation at the outset. As a result, many similar services end up coexisting in parallel to mainstream practice. What is needed, however, is a system-wide transformation that builds upon ongoing quality improvement to better meet patient and population needs [30].

The ‘measuring and communicating outcomes’ theme embodied the importance of systematically tracking eConsult’s progress and growth across Canada and disseminating the results to support spread and scale-up. These objectives are in line with previous research suggesting that a widespread dissemination of eConsult-like programmes will occur only when their positive impact is obvious and the quality of care delivery is acceptable to all populations, including the vulnerable ones [31]. The authors further propose that eConsult’s successful spread and scale-up will depend on the collection and publication of data to comprehensively demonstrate its value in terms of reach, effectiveness, and adoption and should include the consumer/patient perspective. Routine evaluation at the patient level is currently not built into the system since it was developed primarily as a communication platform for providers. However, we have conducted a study where we interviewed patients for whom eConsult was completed by their PCP about their experience [10].

Given the growing importance of collecting patient-centric outcomes in order to ensure delivery of care that meets their needs (value-based healthcare), we have been exploring with our patient partners how this could be done. In the meantime, we have focused on increasing awareness of the service by creating patient-focused information materials (e.g. brochures) designed together with our patient partners and distributing them in several clinics offering eConsult. The feedback received from the clinics has been very positive.

These points are especially important since it is well known that innovations are not self-spreading or self-replicating, and the existing evidence indicates that “*diffusion is an atypical outcome*” [2]. A receptive learning health system, where the emphasis is on generating and applying the best evidence to drive and ensure innovation that is patient centred and focused on high quality, safety and value, is more likely to be in a position of implementation of such a programme [32]. The ultimate goal is for the system to best meet the needs of all Canadians.

### Strengths and limitations

Our study has several strengths. We had a broad representation of different stakeholders across Canada, including patient partners, involved in the data analysis, which allowed for the integration of multiple perspectives and strengthened the quality of the analysis and interpretation. The limitations characteristic to the conduct of qualitative research must also be acknowledged, especially participants’ personal biases and idiosyncrasies. The meeting attendees, our working group and those who conducted the analysis consisted of individuals interested in and hence very supportive of the national spread and scale-up of eConsult. Despite a broad representation of various stakeholders, generalisability can be considered limited in the vast Canadian healthcare context resulting from the country’s 13 unique provincial and territorial jurisdictions, creating a ‘complex labyrinth’ of pathways for innovators. From a methodological standpoint, in small group discussions, we were not able to determine the origin of specific quotations in terms of the type of participant (e.g. patient, researcher, administrator, manager), limiting our ability to identify any differences in opinion between groups.

## Conclusion

Efforts to promote innovation in healthcare are more likely to succeed if they are based on an understanding of the forces that drive the spread and scale-up of innovation. Our in-depth analysis of the discussion surrounding eConsult’s spread and scale-up revealed four themes that reflect strategies for successful spread and scale-up (1) matching the service as the ‘solution’ to the population needs; (2) engaging stakeholders who can action the project in their respective domains (circles of influence); (3) harnessing existing policy, system and implementation enablers; and (4) systematically tracking eConsult’s progress and growth across Canada and disseminating the results. Further research is needed to develop and strengthen the conceptual and applied foundations of the spread and scale-up of healthcare innovations, especially in the context of emergent learning health systems across Canada and beyond.

## Supplementary information

**Additional file 1.** National Forum Agenda.

**Additional file 2.** National Forum – List of transcripts, showing which of the transcribed transcripts were analyzed.

## Data Availability

The datasets created and/or analysed during the current study are available from the corresponding author on reasonable request.

## Abbreviations

PCP: primary care provider
eConsult: electronic consultation
BASE: Building Access to Specialists through eConsultation
