# Supplementary material for: Key factors for national spread and scale-up of an eConsult innovation
Source: Health Res Policy Syst. 2020 Jun 3;18:57. doi: 10.1186/s12961-020-00574-0 (PMC7268606; doi:10.1186/s12961-020-00574-0)
Supplement: Supplementary file 1 — Additional file 1. National Forum Agenda. [file 12961_2020_574_MOESM1_ESM.docx]

**Appendix A.** National Forum Agenda

| **Start** | **End** | **Item Details** |
| --- | --- | --- |
| 9:00 | 9:15 | **Welcome / housekeeping**  *Dr. Clare Liddy* |
| 9:15 | 9:30 | **Opening remarks**  Setting the stage and understanding eConsult through the lens of the Royal College of Physicians and Surgeons of Canada  *Mrs. Danielle Frechette / RCPSC* |
| 9:30 | 9:45 | **Understanding best practices**  This presentation will introduce the concept of best practices and outline the key objectives for the day. Time for questions and answers will be included  *Dr. Erin Keely* |
| 9:45 | 10:15 | **Understanding the Canadian landscape**  Overview presentation of the Canadian landscape of services to improve access to specialist advice, including RACE (urgent access), eConsult (non urgent access) and eReferral (face-to-face visits), and the intersection amongst these approaches  *Multiple presenters representing the stakeholder groups* |
| 10:15 | 10:30 | **Refreshment break and networking** |
| 10:30 | 11:00 | **Reflections from patients on eConsult as patient-centred practice**  *Patient partner presenters* |
| 11:00 | 12:15 | **Concurrent workshop sessions to develop best practice recommendations**  *Multiple facilitators chosen for their expertise in the topic area* |
|  |  | **A1: eReferral and eConsult integration**  This session will focus on the intersection between eConsult and eReferral, including who is the gatekeeper, institutional versus regional programs, organization of specialty services |
|  |  | **A2: Primary care adoption for sustained utilization**  Sustained primary care adoption is key to the success of eConsult/eReferral and RACE. This session will focus on identifying key factors, such as ease of use, EMR integration, role of remuneration, then identifying what works and what are the expectations for best practices. |
|  |  | **A3: Considerations for populations with complex circumstances**  eConsult/eReferral/RACE are all services that can improve access to people with complex circumstances such as geography (rural/remote), location (long-term care), indigenous, low socio-economic status. Discussion will centre on key enablers to ensure equitable access which could include the role of nurses in remote communities, generalist participation especially in small communities, patient consent processes and interjurisdictional licensing. |
| 12:15 | 1:00 | **Lunch and networking** |
| 1:00 | 1:30 | **Reflections on the morning sessions -** *Dr. Erin Keely*   1. What are your key recommendations for best practices in this area? 2. What gaps still exist that must be addressed? 3. Who are the key external stakeholders to review best practices document? |
| 1:30 | 2:45 | **Concurrent workshop sessions to develop best practice recommendations**  *Multiple facilitators chosen for their expertise in the topic area* |
|  |  | **B1: Effective Governance**  Effective governance contributes greatly to the success of any systemic change. How do we ensure the right people in the right roles are at the table? How do we engage patients in meaningful ways? How are interactions with providers and quality management managed? Who should have access to what data? Participants will have the ability to contribute to building recommendations on strategies for effective governance of these technology-enabled specialist access initiatives. |
|  |  | **B2: Integration of eConsult/eReferral Workflows into EMRs**  Questions abound as to the best approach to integrate eConsult and eReferral workflows into EMRs. The discussion will focus on key considerations including who should be the gatekeeper, the types of models and processes, the role and responsibility of vendors, (not-for profit and for-profit), records retention and privacy. |
|  |  | **B3: Considerations for specialist’s participation**  The role of the specialist in the eConsult/eReferral workflow needs exploring and recommendations developed. Should all specialists participate in all services? What is needed when reorganizing access to specialist services? What competencies are needed? How are key competencies identified and linked to Continuing Medical Education? |
| 2:45 | 3:00 | **Refreshment break and networking** |
| 3:00 | 3:30 | **Report back from small group sessions**  1. What are your key recommendations for best practices in this area?  2. What gaps still exist that must be addressed?  3. Who are the key external stakeholders to review best practices document? |
| 3:30 | 3:45 | **Reflections from the RCPSC Perspective**  *Dr. Douglas Heddon* |
| 3:45 | 4:15 | **Developing a work plan with participant commitments**  We will ask participants to choose to commit to action to help move the development of best practices forward. We anticipate a lively discussion on the best way forward as we move to build best practices that leverage the work done during the day.  *Dr. Erin Keely & Mr. Neil Drimer* |
| 4:15 | 4:30 | **Wrap up and next steps**  Summary of the day’s deliberations and developing consensus on next steps.  *Dr. Clare Liddy* |
