# Supplementary material for: Key factors for national spread and scale-up of an eConsult innovation
Source: Health Res Policy Syst. 2020 Jun 3;18:57. doi: 10.1186/s12961-020-00574-0 (PMC7268606; doi:10.1186/s12961-020-00574-0)
Supplement: Supplementary file 2 — Additional file 2. National Forum – List of transcripts, showing which of the transcribed transcripts were analyzed. [file 12961_2020_574_MOESM2_ESM.docx]

**Appendix B.** National Forum – List of transcripts, showing which of the transcribed transcripts were analyzed.

|  | Session | Transcribed | Analyzed |
| --- | --- | --- | --- |
| 1 | Welcome Remarks & Introductory Comments | Y | N |
| 2 | Overview of eConsult Research Evidence and Implementation Successes | Y | N |
| 3 | National Implementation | Y | N |
| 4 | Education Projects | Y | N |
| 5 | Specialist Perspectives | Y | N |
| **6** | **Provincial Updates Alberta** | Y | Y |
| **7** | **Key Enablers of Implementation** | Y | Y |
| 8 | **Provincial Updates Ontario** | Y | Y |
| **9** | **Provincial Updates Newfoundland** | Y | Y |
| **10** | **Provincial Updates Manitoba** | Y | Y |
| **11** | **Provincial Updates Quebec** | Y | Y |
| **12** | **Patient Panel Reflections** | Y | Y |
| 13 | Small Group Sessions  1 – Considerations for a national service  2 – Integrating eConsult into clinic workflows  3 – Sharing engagement and communication strategies  4 – Leveraging eConsult for Continuing Professional Development  5 – Special Population Needs | Y  Y  Y  Y  Y | N  N  N  N  N |
| **14** | **Small Group Session Report Back** | Y | Y |
| 15 | Table Top Hot Topics Session: Practical tools for eConsult spread and scale | Y | N |
| **16** | **Table Top Session Practical Tools Report Back** | Y | Y |
| 17 | Table Top Hot Topics Session: Policy Briefs | Y | N |
| **18** | **Table Top Session Policy Briefs Report Back** | Y | Y |
| **19** | **National Leaders Presentations and Next Steps** | Y | Y |
